# Biosynthetic Pathway of psi, psi-Carotene from *Streptomyces* sp. VITGV38 (MCC 4869)

**DOI:** 10.3389/fmicb.2025.1548894

**Published:** 2025-04-01

**Authors:** Veilumuthu Pattapulavar, Sathiyabama Ramanujam, Manoj Sekaran, Rajasekaran Chandrasekaran, Shweta Panchal, John Godwin Christopher

**Affiliations:** ^1^Department of Biomedical Sciences, School of BioSciences and Technology, Vellore Institute of Technology, Vellore, Tamil Nadu, India; ^2^Department of Science and Humanities, Karpagam Academy of Higher Education, Coimbatore, India; ^3^Department of Biotechnology, School of BioSciences and Technology, Vellore Institute of Technology, Vellore, Tamil Nadu, India; ^4^Department of Integrative Biology, School of BioSciences and Technology, Vellore Institute of Technology, Vellore, Tamil Nadu, India

**Keywords:** secondary metabolite, *Streptomyces* sp. VITGV38, pigment, biosynthetic gene cluster, psi, psi-carotene, antiSMASH

## Abstract

**Introduction:**

Endophytic *Streptomyces* play a crucial role in plant-microbe interactions, often exhibiting beneficial biological activities, including the production of bioactive secondary metabolites. This study aimed to characterize the carotene biosynthetic pathway of a newly discovered *Streptomyces* sp. VITGV38, isolated from tomato (Lyc*opersicon esculentum*).

**Methods:**

The strain (*Streptomyces* sp. VITGV38, MCC4869) was cultured in starch casein broth, and its metabolite profile was analyzed using Gas Chromatography-Mass Spectrometry (GC-MS). Whole-genome sequencing was performed using the Illumina platform, and the biosynthetic gene clusters (BGCs) were identified using antiSMASH.

**Results:**

Metabolite analysis revealed the presence of pigmented compounds, including psi, psi-carotene, detected at a retention time of 25.094, constituting 1.26% of the crude extract. Whole-genome sequencing uncovered an 8.27 Mb genome encoding 26 distinct secondary metabolite biosynthetic gene clusters. Notably, scaffold 26.3 was identified as a terpene biosynthetic cluster, accounting for 62% of the total secondary metabolite content and associated with carotenoid and β-carotene production.

**Discussion:**

These findings highlight the biotechnological potential of *Streptomyces* sp. VITGV38 for sustainable microbial production of carotenoids, offering an eco-friendly alternative to synthetic pigments. This study provides valuable insights into microbial carotenoid biosynthesis and its potential industrial applications.

## Introduction

Pigments play a fundamental role in nature, contributing to the vibrant colors of organisms while serving crucial biological functions. Among microbial pigments, carotenoids stand out due to their diverse applications in health, industry, and ecology. These natural pigments, ranging from yellow to deep red, are widely recognized for their antioxidant properties, membrane stabilization, and photoprotective roles (Maoka, 2020). Carotenoids are extensively utilized in food, feed, nutraceutical, and pharmaceutical industries, with the global market for astaxanthin alone projected to reach $2.57 billion by 2025 (Chen et al., 2021).

Endophytic *Streptomyces* species have recently garnered significant attention due to their pivotal roles in symbiotic relationships, particularly in association with plants (Cao et al., 2004). While *Streptomyces* species are renowned for their antibiotic-producing capabilities, their potential as pigment producers remains relatively underexplored. Endophytic *Streptomyces*, in particular, offer a unique and untapped reservoir for pigment biosynthesis, as their symbiotic interactions with plants may influence secondary metabolite production, including carotenoids. Carotenoids, a class of natural pigments with antioxidant properties, play essential roles in biological systems. Beyond their function as pigments, carotenoids also serve as structural molecules, integrating into lipid membranes and modulating membrane fluidity (Maoka, 2020). The diverse applications of carotenoids in food, feed, nutraceutical, and pharmaceutical industries have fueled a surge in global demand (Chen et al., 2021).

Carotenoid of interest is psi, psi-carotene, also known as lycopersene, is found in various red-colored fruits and vegetables, including carrots. This psi, psi-carotene exhibits a spectrum of beneficial effects, including antioxidant, antimutagenic, antiproliferative, and antibacterial properties (Khoo et al., 2011). While *Streptomyces* species are renowned for their role as prolific antibiotic producers, their capacity to synthesize carotenoid pigments has also been documented. However, the production of psi, psi-carotene by *Streptomyces* has not been previously reported for any endophytic *Streptomyces* species.

The biosynthesis of carotenoids is a complex process governed by intricate pathways involving various enzymes and regulatory genes. Carotenoid synthesis pathways have been extensively studied in diverse organisms, ranging from bacteria and fungi to higher plants (Yuan et al., 2015). *Streptomyces* species, known for their diverse secondary metabolite production capabilities, often harbor cryptic gene clusters responsible for the synthesis of novel compounds. Despite the cryptic nature of these gene clusters under standard laboratory conditions, bioinformatics tools have facilitated their identification and characterization (Scherlach and Hertweck, 2021).

In our previous study, we sequenced the complete genome of *Streptomyces* sp. VITGV38, establishing a foundation for investigating its biosynthetic capabilities (Veilumuthu et al., 2022; Veilumuthu et al., 2023). Building upon this work, our current study aims to elucidate the biosynthetic pathway of psi, psi-carotene in *Streptomyces* sp. VITGV38 (MCC 4869) using bioinformatics tools such as antiSMASH and MIBiG. Specifically, we focus on identifying core biosynthetic genes and regulatory elements involved in psi, psi-carotene biosynthesis. These findings highlight the genetic distinctiveness of this strain and its potential for secondary metabolite production. This study presents a comprehensive genomic and metabolic characterization of *Streptomyces* sp. VITGV38, shedding light on its ability to produce psi, psi-carotene and expanding our understanding of carotenoid biosynthesis in endophytic *Streptomyces* species. The results contribute to the growing body of knowledge on microbial carotenoid production and its potential industrial applications.

## Materials and methods

### Identification and characterization of *Streptomyces* sp. VITGV38

The strain *Streptomyces* sp. VITGV38 was identified using phenotypic and genotypic characterization [11]. The preparation of the samples for scanning electron microscopy was done according to Veilumuthu and Christopher (2022a, b, c). The strain VITGV38 was grown in starch casein agar (SCA) for 7 days. These imaging techniques seamlessly unveiled the intricate morphological attributes of the strain, rendering a vivid depiction of its distinctive characteristics.

### Genome characterization, annotation, and gene cluster detection

Genomic DNA was extracted from *Streptomyces* sp. VITGV38 grown on ISP2 medium for 15 days according to Yang et al. (2023). The DNA libraries, primed for sequencing, were adeptly prepared utilizing the TruSeq Nano kit. The subsequent sequencing procedure was conducted using the Illumina NextSeq 500 platform, with Eurofins Genomics Private Ltd. Bangalore. This approach generated paired-end reads with a length of 150 bp, ensuring that the vast majority—more than 20—exhibited Phred scores of Q30 or higher. After the read acquisition, a trimming process was executed using a sliding window of 10 bp, employing a threshold of 20. The processed reads were then orchestrated into cohesive scaffolds via the SPAdes assembler (v-3.13.0). These scaffolds were judiciously juxtaposed with homologous sequences to discern the most closely related organism(s).

The genome of VITGV38, annotated using Prokka (version 1.12) and KEGG Annotation of protein-coding and RNA genes from the genome were identified in the samples using the final assembled draft genome. The process of gene prediction was performed by Prokka (version 1.12) (Seemann, 2014). The pathway annotation of predicted genes was carried out against the curated KEGG GENES database using KAAS (KEGG Automatic Annotation Server; http://www.genome.jp/kegg/ko.html). The genome is uploaded to NCBI as *Streptomyces shanivit* under BioProject ID PRJNA750621. The circular genome was constructed using the server CGView (Grant and Stothard, 2008). Phylogenetic analysis was performed using the 16 s ribosomal RNA sequences (collected from NCBI) using MegaX (Kumar et al., 2018). The biosynthetic clusters involved in the synthesis of secondary metabolites were analyzed using antiSMASH 6.0 (Blin et al., 2021) and BAGEL 4 (van Heel et al., 2018). This adept software facilitated the identification of biosynthetic gene clusters, functional genes, and secondary metabolites with precision according to Veilumuthu et al. (2024).

### GC-MS analysis of crude extracts of *Streptomyces* sp. VITGV38

To investigate the metabolic profile of *Streptomyces* sp. VITGV38, the strain was cultured in starch casein medium supplemented with 3% sodium chloride and incubated on a shaker for 21 days. After incubation, the culture broth (30 liters total) was centrifuged to separate the pellet and the supernatant. Metabolites from the supernatant were extracted using ethyl acetate in a 1:1 (v/v) ratio. The extract was concentrated using a rotary evaporator and subsequently analyzed via gas chromatography–mass spectrometry (GC-MS) to identify volatile and semi-volatile bioactive compounds (Veilumuthu and Christopher, 2022a, 2022b, 2022c). GC-MS analysis was performed using a Thermo Scientific Clarus 680 GC instrument equipped with an Elite-5MS fused silica column (30 m × 0.25 mm ID × 250 μm df, 5% biphenyl, 95% dimethylpolysiloxane). The carrier gas was helium at a constant flow rate of 1 mL/min. The injector temperature was maintained at 260°C throughout the chromatographic run. The mass spectrometer was operated in electron ionization mode, and compound identification was performed by comparing spectral data with the NIST mass spectral database. GC-MS was chosen due to its high sensitivity in detecting low-molecular-weight, volatile, and semi-volatile compounds, which may contribute to the bioactivity of the crude extract. This method allows for the identification of pigment-associated metabolites such as terpenes and carotenoids, which are central to this study.

### HPLC analysis and fraction collection

To further analyze and fractionate the methanolic extract of *Streptomyces* sp. VITGV38, high-performance liquid chromatography (HPLC) was employed. The analysis was conducted using a Shimadzu HPLC system (CBM-20A system, binary LC-20AP pump, SPD-M20A Photo Diode Array (PDA) detector). Chromatographic separation was achieved using a Shimpack GIST C18 column (250 × 4.6 mm, 5 μm particle size). The mobile phase consisted of a degassed water–methanol mixture, filtered through a 0.25 μm nylon membrane (Merck). A total of 10 μL of the sample was injected via a SIL-20 AC HT Autosampler, and isocratic elution was performed at a flow rate of 1 mL/min. Peak separation efficiency was assessed through chromatographic analysis, and the resulting peaks were examined using LC-Solution tools (Shimadzu Corporation, Kyoto, Japan). HPLC was utilized to isolate and purify specific carotenoid fractions from the crude extract, ensuring a more targeted analysis of bioactive compounds. Unlike GC-MS, which is optimal for volatile compounds, HPLC is better suited for analyzing non-volatile, thermally labile metabolites such as carotenoids.

### LC-HRMS-ESI analysis for chemical profiling

Further structural elucidation of bioactive compounds was performed using liquid chromatography-high-resolution mass spectrometry (LC-HRMS-ESI). The LC-HRMS analysis was conducted using a Waters^®^ Micromass^®^ Q-TOF Micro^™^ mass spectrometer. Data acquisition and processing were conducted using LC-Solution tools (Shimadzu Corporation, Kyoto, Japan). LC-HRMS-ESI was chosen for its superior accuracy in mass determination, enabling precise identification of complex secondary metabolites. This technique complements HPLC by providing molecular weight information and structural insights into pigment-related metabolites.

## Results

**Figure 1:**
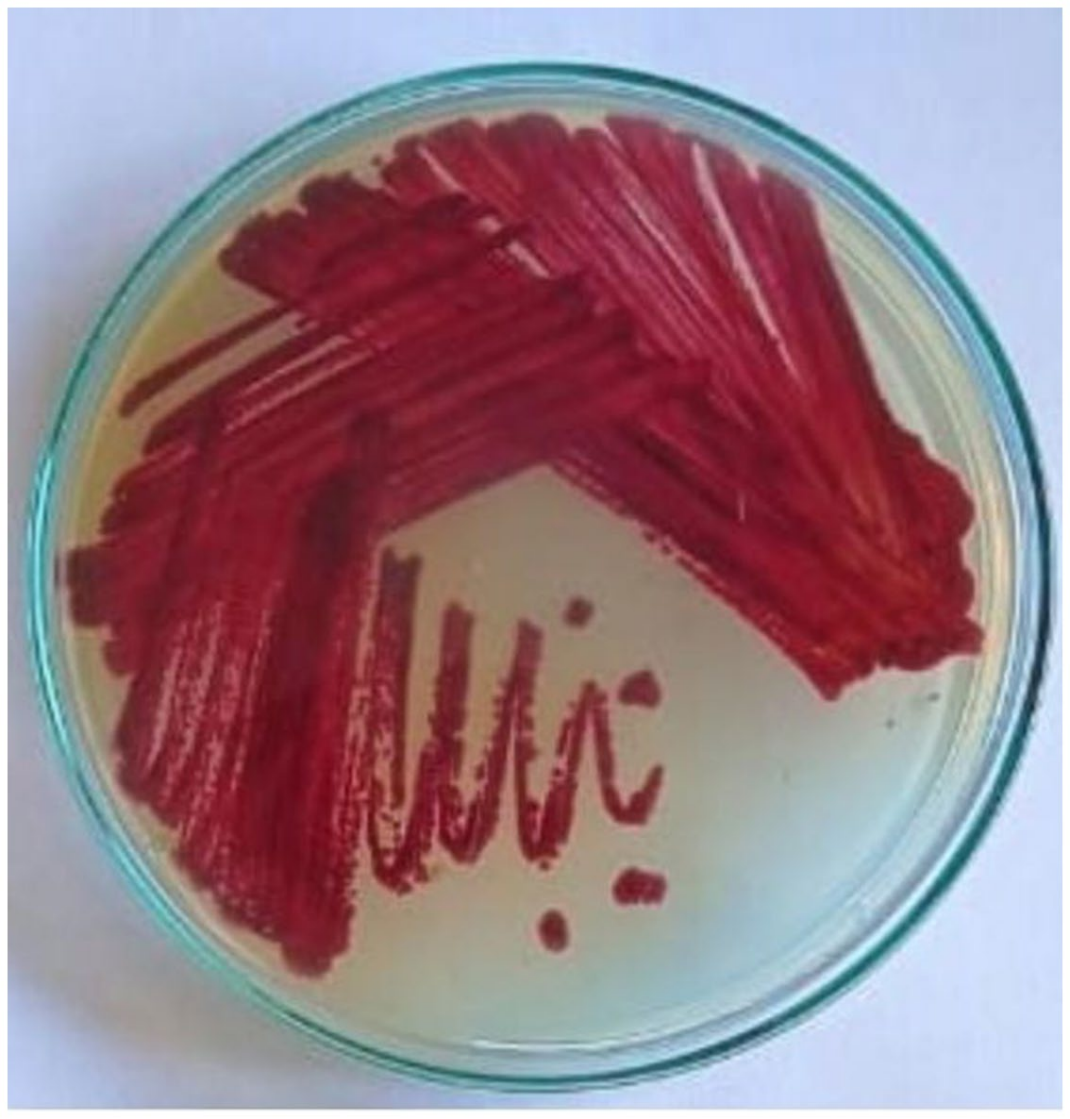
Growth and pigment production of *Streptomyces* sp. VITGV38, grown for 7 days in starch casein agar.

Endophytes are intimately involved in the physiology of the host plant. These endophytes have been shown to provide beneficial attributes to the plant for growth, defense, stress, etc. In this study, we report a *Streptomyces* sp. VITGV38, an endophytic spore forming organism. This strain produces more diffusible pigment in the SCA medium (Figure 1). Phenotypic characterization of *Streptomyces* sp. VITGV38 on ISP2 agar revealed the formation of aerial mycelia with a red substrate mycelium.

### Genome analysis

**Figure 2:**
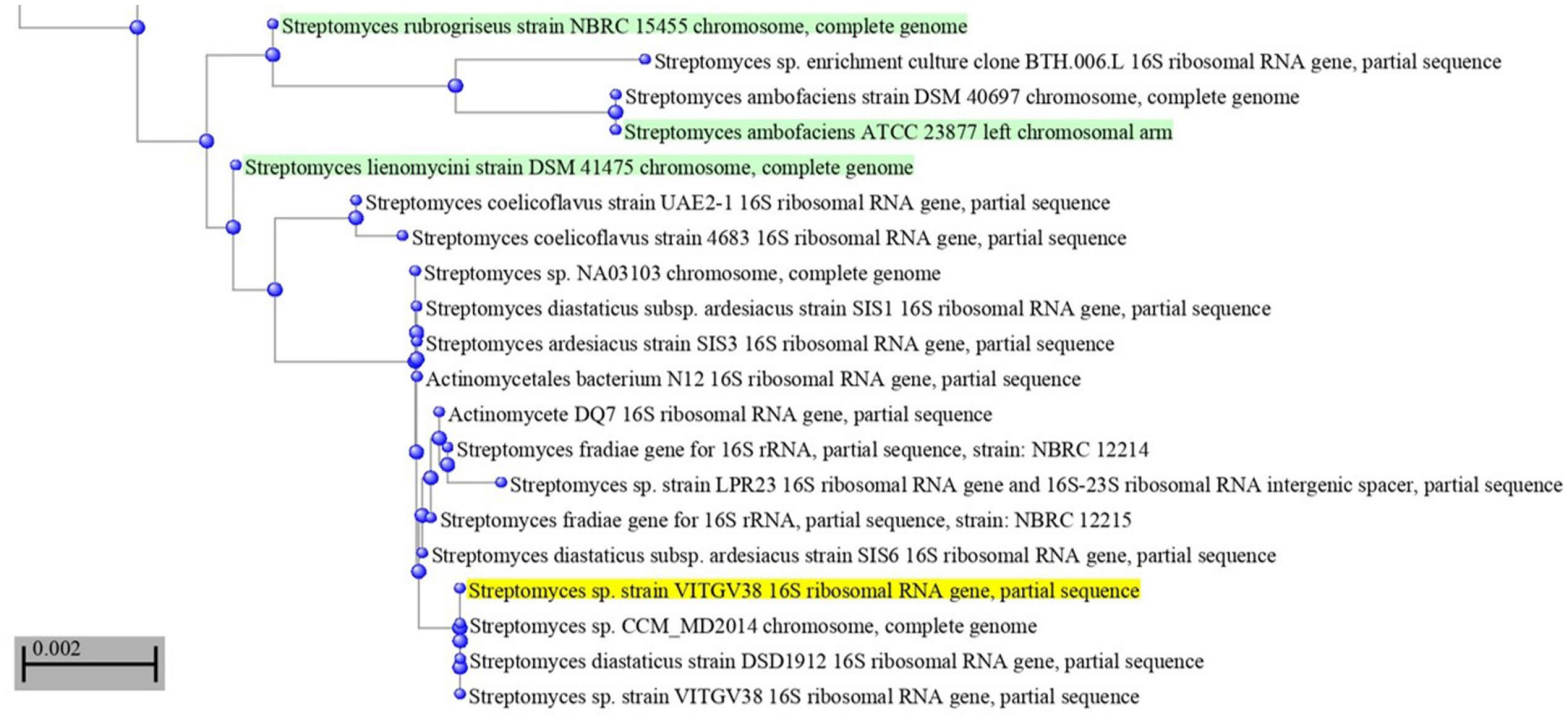
Phylogeny of *Streptomyes* sp. VITGV38. A Neighbor-joining tree based on 16S rRNA gene sequence data, depicting the phylogenetic position of the strain VITGV156 among the recognized type strains of the genus *Streptomyces*. The minimum evolution tree based on genome sequence of recognized strains from the genus *Streptomyces* was constructed blastn database provided pair wise alignment. The strain used in this study was provided in yellow color highlighted.

**Figure 3:**
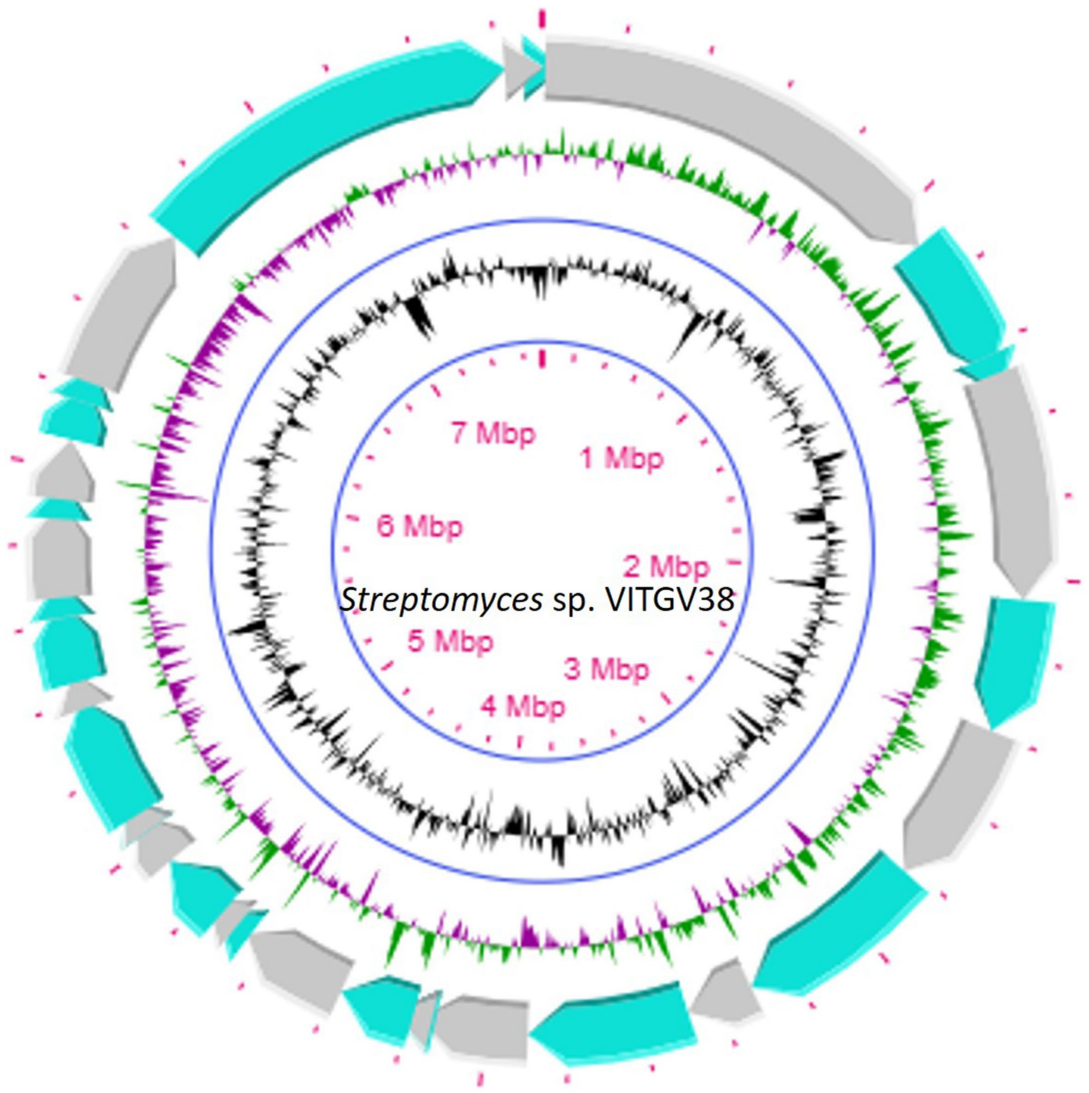
Circular genome map of *Streptomyces* sp. VITGV38, generated using CG View (1.0). The following features are shown (moving from the outermost track to the innermost track); origin of replication positioned: ORF genes in green color, positive and negative GC content skew in green and purple, respectively. GC content (black) and the genome position in the center. The innermost ring displays the overall genome size of *Streptomyces* sp. VITGV38.

This strain was subjected to whole genome sequencing using the Illumina Nextseq500 platform followed by bioinformatics analysis, which revealed its size as 8.27 Mb. Figure 2 shows the circular genome map *Streptomyces* sp. VITGV38. The genome sequencing data (GC content, genome size, total No. of genes, dDDH, and ANI) indicated that it is a species belonging to the genus *Streptomyces* (Table 1). The phylogenetic position of *Streptomyces* sp. VITGV38 was determined using a Neighbor-Joining tree based on 16S rRNA gene sequence data. The analysis revealed its clustering among the recognized type strains of the genus *Streptomyces*, indicating its taxonomic placement within this genus. Furthermore, a minimum evolution tree was constructed based on genome sequences of recognized *Streptomyces* strains using the BLASTn database, which provided pairwise alignment results. The strain used in this study was specifically highlighted in yellow to distinguish its placement in the phylogenetic tree (Figure 3). The OrthoANIu analysis demonstrated that *Streptomyces* sp. VITGV38 exhibited a 92.15% similarity with *Streptomyces diastaticus* strain SID7513. Additionally, the digital DNA–DNA hybridization (DDH) value between *Streptomyces* sp. VITGV38 and *Streptomyces diastaticus* strain SID7513 was found to be 46.90%. These values suggest a close phylogenetic relationship between the two strains, further supporting the taxonomic classification of *Streptomyces* sp. VITGV38.

**Table 1:** General features of *Streptomyces* sp. VITGV38 genome.

S. No	Features	*Streptomyces* sp. VITGV38
1	Overall genome size (bp)	8.27 Mb
2	The total number of bases sequenced	7,715,899
3	GC content (%)	72.28
4	Total genes	6,938
**5**	**OrthoANIu value** *Streptomyces diastaticus* Strain SID7513 (%)	92.15
**6**	**DDH value** *Streptomyces diastaticus* Strain SID7513 (%)	46.90
7	Protein coding genes	6,812
8	Genes with no blast hit	158
9	rRNA operons	5
10	tRNA operons	80

### GCMS analysis of VITGV38

**Figure 4:**
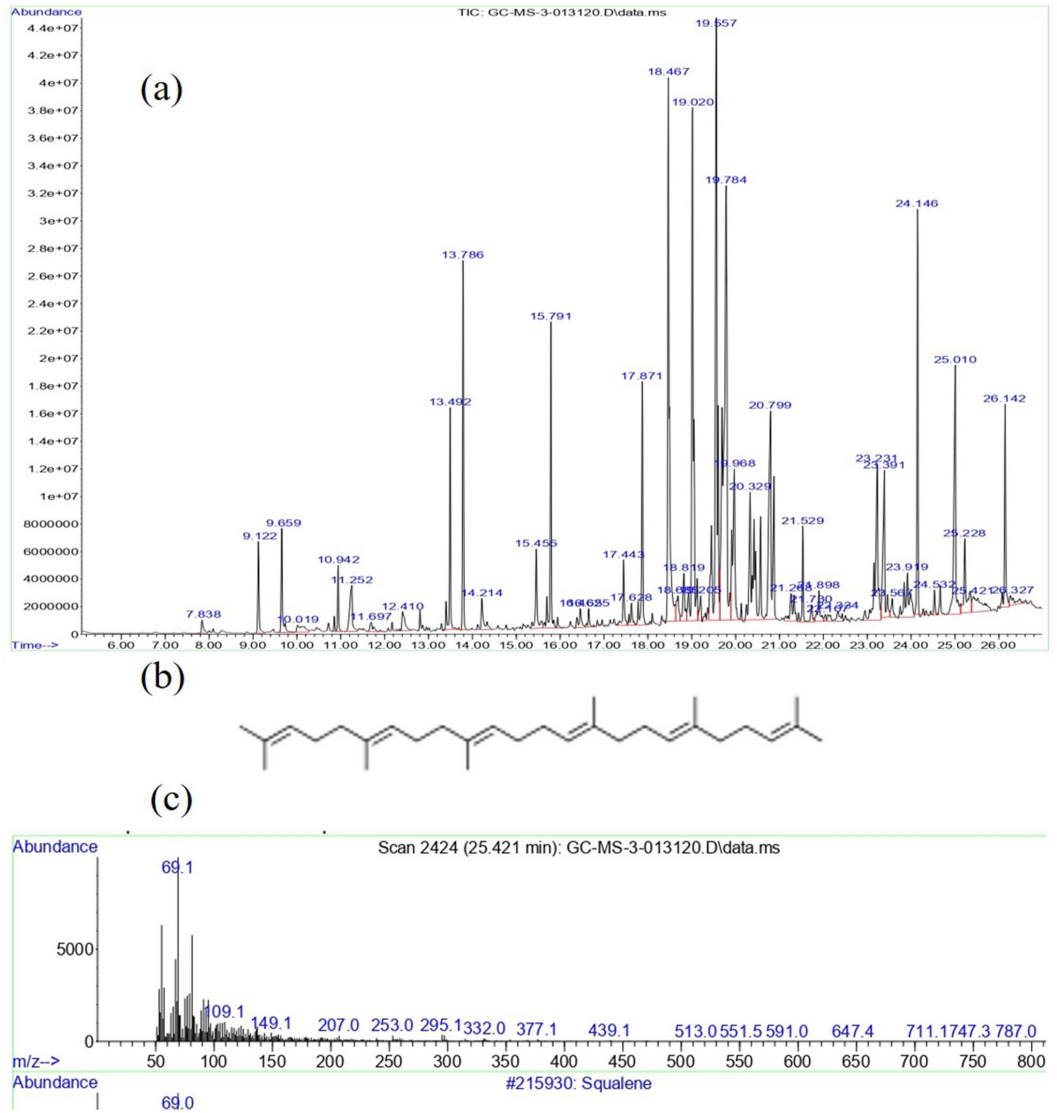
**(A)** GC-MS chromatogram of Streptomyces sp. VITGV38 ethyl acetate extract. **(B)** Mass spectrum of psi, psi-carotene. **(C)** Molecular structure of psi, psi-carotene.

Gas chromatography-mass spectrometry (GC-MS) analysis is a powerful analytical technique used in chemistry to identify and quantify components in a mixture. GC-MS is valued for its high sensitivity, specificity, and ability to analyze complex mixtures. It is widely used in research and industries for its versatility and reliability in chemical analysis. In the current investigation, the VITGV38 was obtained from tomato plants and subsequently cultivated under optimized laboratory conditions to induce the production of metabolites. These metabolites were then extracted using ethyl acetate as the solvent. Following extraction, the resulting material was condensed, yielding VITGV38 metabolite extract. The crude extracts obtained were then subjected to GCMS analysis. Figure 4A shows the GCMS analysis of VITGV38. A total of 45 compounds were identified from VITGV38 extracts. Supplementary Table S1 represents the GCMS identification of compounds from VITGV38. Among the compounds examined, the detection of psi, psi-carotene (from VITGV38) sourced from *streptomycetes* inhabiting tomato plants represents a novel finding.

The GCMS analysis of the culture extract in ethyl acetate showed 45 peaks, with psi, psi-carotene at a retention time of 25.094 min constituting 1.26% of the total crude extract (Figures 4B,C). Comparing this data with the NIST mass spectrophotometry database, confirmed the presence of psi, psi-carotene in the extract. To our knowledge, no other *Streptomyces* species has been shown to produce this pigment and this is the first report of an endophytic *Streptomyces* producing psi, psi-carotene. Consequently, we aimed to isolate these metabolites from the respective extracts owing to their promising pharmaceutical significance. Utilizing preparative HPLC, we effectively isolated psi, psi-carotene, and their molecular weights were subsequently confirmed through LC-HRMS analysis.

### HPLC and LCHRMS analysis of isolated compounds

**Figure 5:**
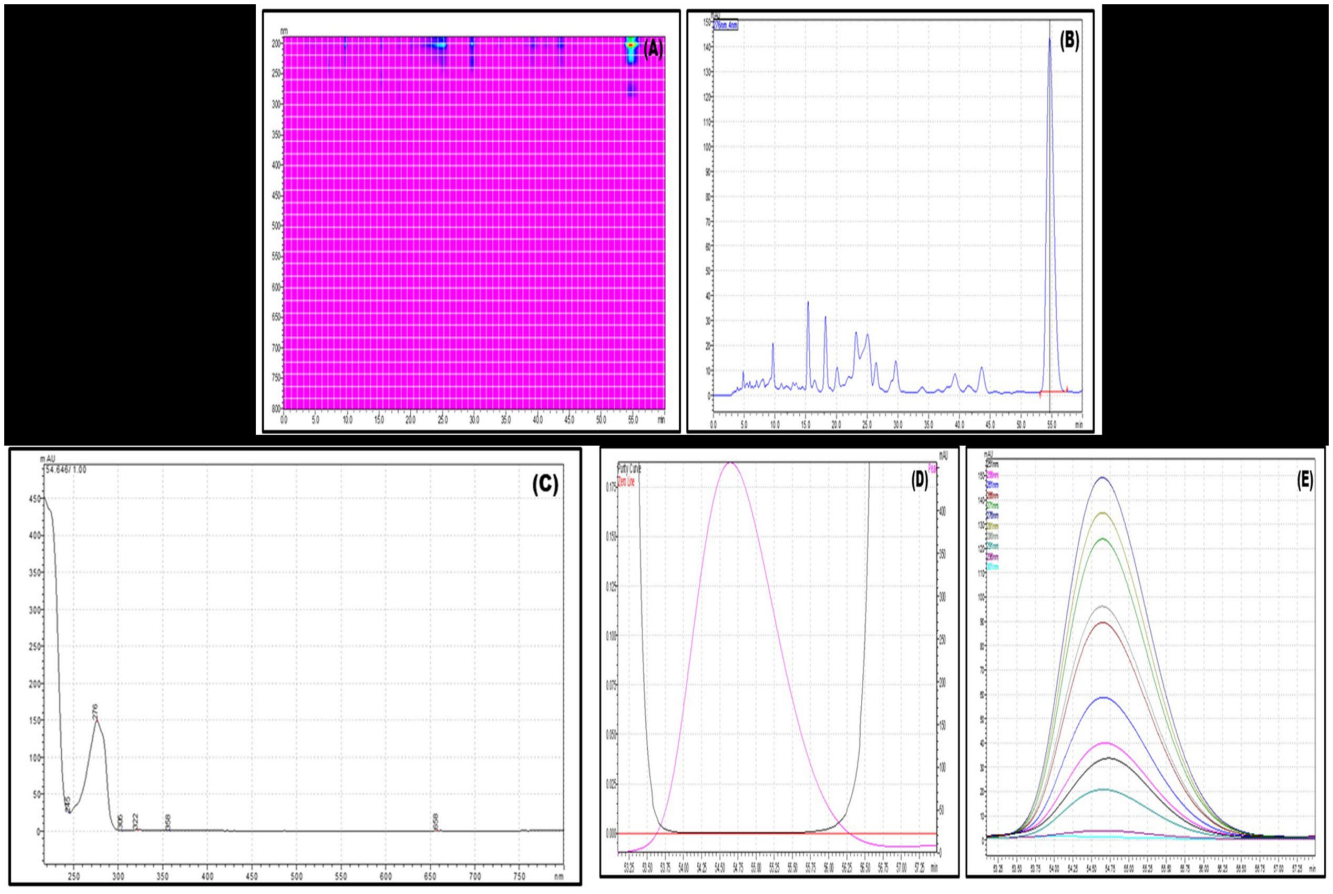
A RP-HPLC separation of crude extract of 38. **(A)** 3D counter view. **(B)** Chromatogram of the crude extract. **(C)** UV spectrum at retention time (RT) 54.6 min. **(D)** Purity index of the peak at RT 54.6. **(E)** Peak profile of the targeted compound.

Several isocratic methods were experimented with, employing different ratios of methanol–water and acetonitrile-water mixtures, along with varying flow rates, aiming to optimize chromatographic conditions for separating these compounds from the crude extract of VITGV38. The most effective separation was achieved using a mobile phase comprising methanol and water in a 30:70 (v/v) ratio, with a 1 mL/min flow rate. High-intensity peaks were identified by comparing them to the retention time (RT) of 54.6 and the maximum absorbance wavelength (*λ*_max_) of 276 nm for the targeted compound. The eluted fraction exhibited a peak profile with satisfactory resolution and a purity index 0.99, indicating no interference with the targeted compounds (Figure 5).

**Figure 6:**
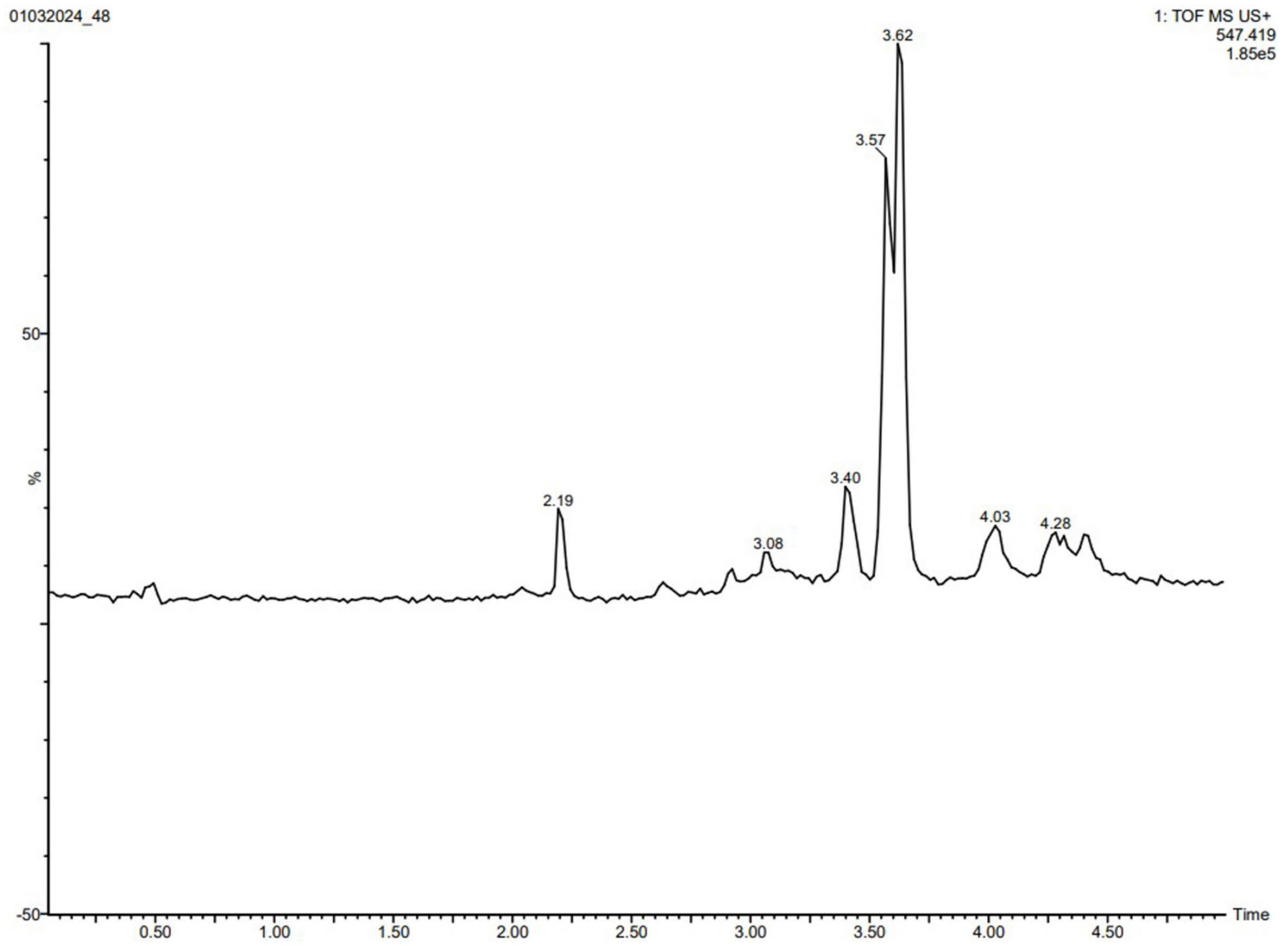
LC-MS/MS chromatogram of the secondary metabolite of *Streptomyces* sp. VITGV38. The peak corresponds to the bioactive molecule psi, psi-carotene (peak at 3.637, *m/z* = 546.5165).

**Figure 7:**
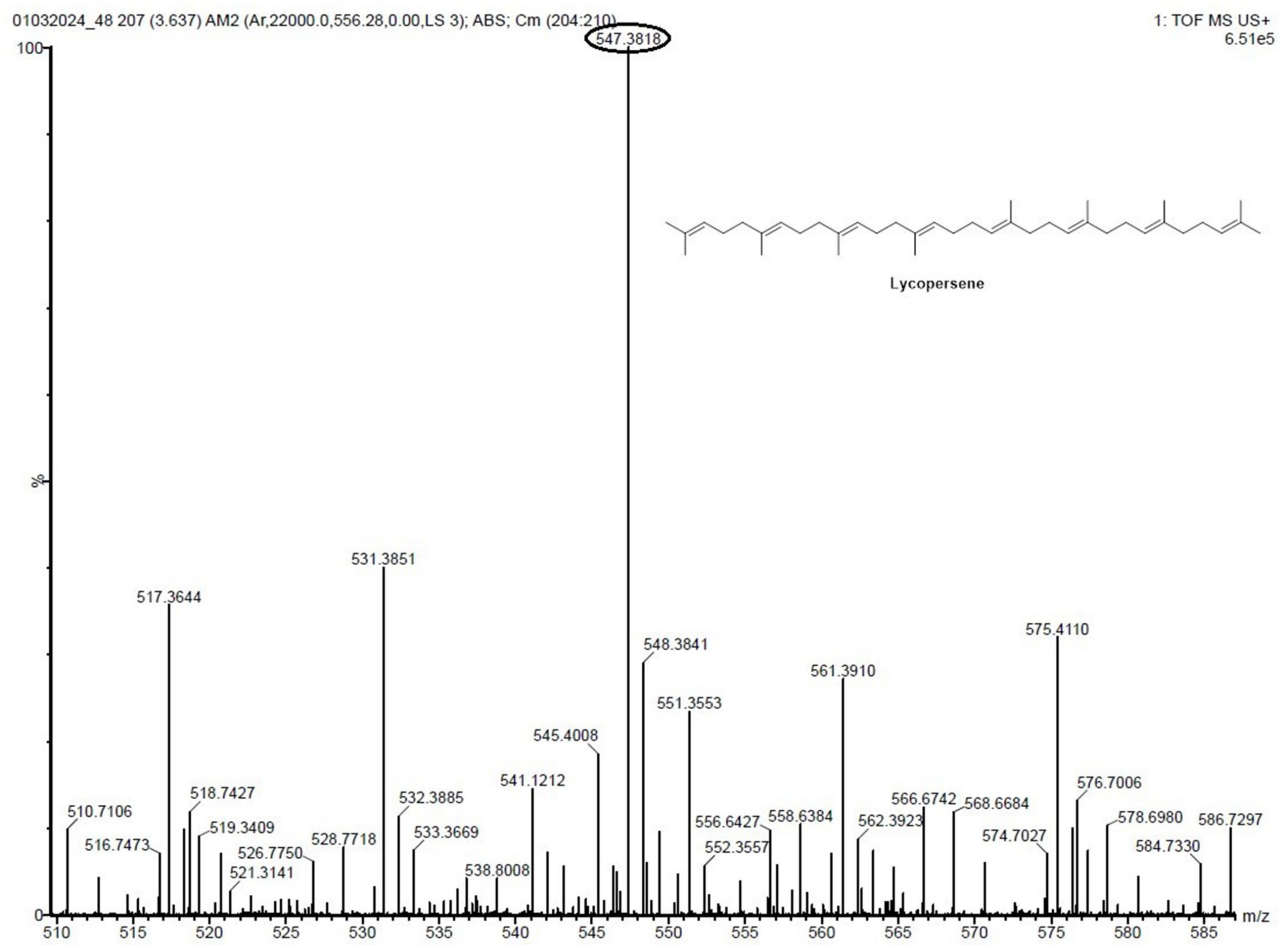
LC-HRMS-ESI spectrum of the compound psi, psi-carotene retention times, names, molecular formulas, molecular masses, and obtained masses of analyzed compounds.

To swiftly identify these metabolites from a limited number of samples, we conducted LC-HRMS analysis on both the VITGV38 crude extract (Figure 6). The LC-HRMS data were scrutinized utilizing *m*/*z*, retention time, and molecular formula, with additional databases utilized to search for and assign formulas and compound structures. Following evaluation and interpretation, the compound identified at a retention time RT value of 3.63 (Figure 7), the identified compound was psi, psi-carotene. Details regarding the RT, molecular formula, and molecular mass of these identified compounds are provided in Table 2.

**Table 2:** Retention times, names, molecular formulas, molecular masses, and obtained masses of analyzed compounds.

S. No.	RT (min)	Name and molecular formula	Molecular mass (g/mol)	Obtained mass (g/mol)
1	3.63	psi, psi-carotene (C_40_H_66_)	546.5165	547.3818 [M + H]

### Bioinformatic conformation of psi, psi-carotene

**Figure 8:**
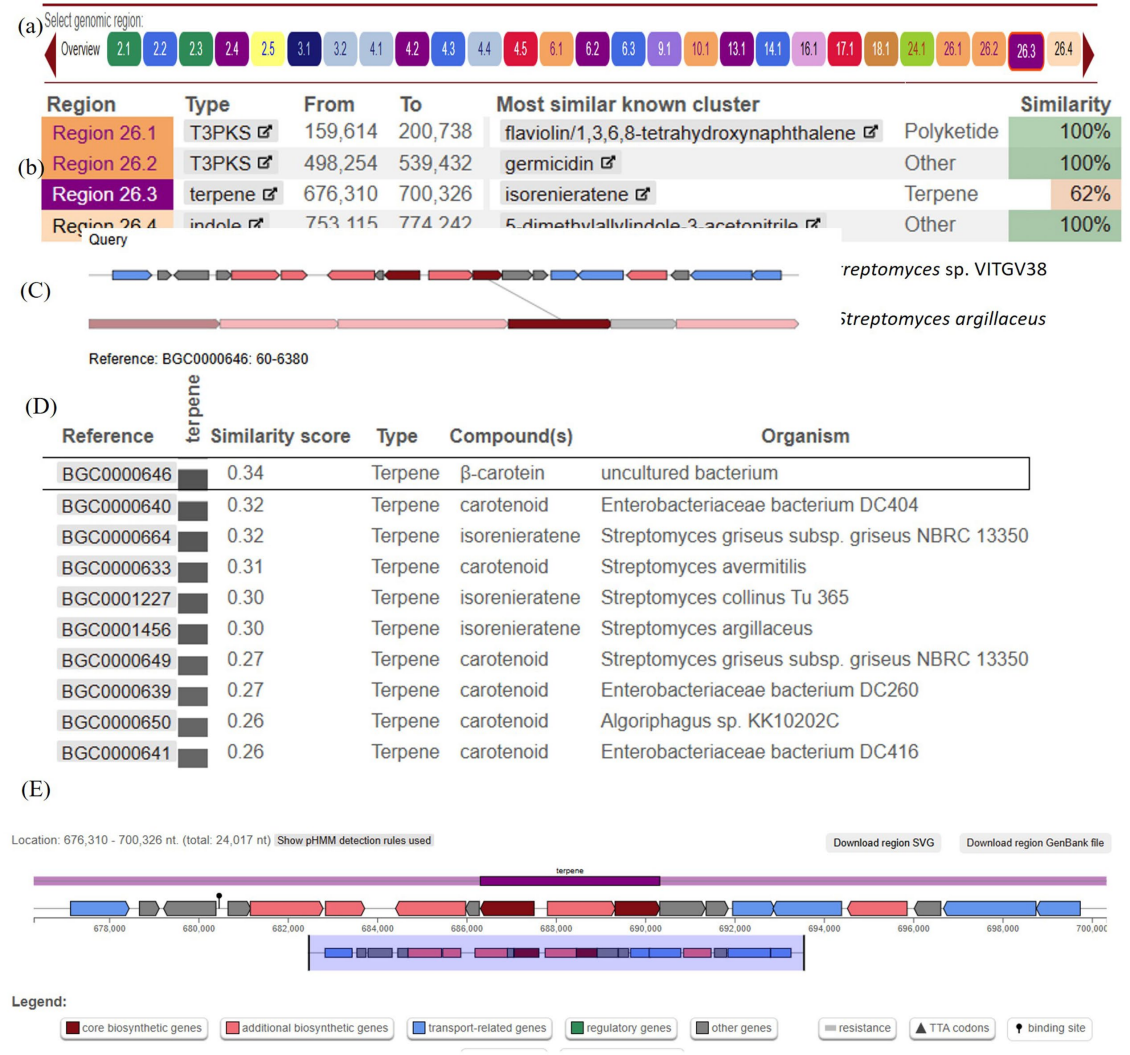
The overall genetic architecture of (isorenieratene) terpene gene cluster in *Streptomyces* sp. VITGV38. **(A)** Overall view of biosynthetic gene clusters. **(B)** Specific region for terpene clusters. **(C)** The comparison between the proposed hopene gene cluster (dark red gene) in strain *Streptomyces* sp. VITGV38 and the proposed identified cluster of isorenieratene in *Streptomyces argillaceus* with a similarity score of 0.30 on the MIBiG website. **(D)** The proposed terpene gene cluster in region 23.6 from strain *Streptomyces* sp. VITGV38 genome contains; 2Fe-2S ferredoxin, phytoene/squalene synthase, phytoene desaturase, geranylgeranyl pyrophosphase synthase, RNA polymerase Sigma-24 subunit, lycopene cyclase, methyltransferase and isorenieratene synthase. **(E)** Represents the predicted genes involved in isorenieratene biosynthesis based on the genes overview on the antiSMASH website.

The psi, psi-carotene analysis module is fully integrated in version 7.0.1 of antiSMASH (released year 2023). When antiSMASH identifies a carotenoid BGC, the new module is triggered to provide further information on the identified BGC. antiSMASH uses a rule-based detection algorithm to identify clusters based on core biosynthetic enzymes. We also identified 26 biosynthetic gene clusters (BGCs) in the genome of VITGV38 (Table 3 and Figure 8A). Based on the *in silico*, analysis, the *Streptomyces* sp. VITGV38 genome revealed five terpene gene clusters (Figure 8B). Among the five terpene clusters, the region 26.3 codes isorenieratene, this isorenieratene has 62% similarity with the isorenieratene from *Streptomyces argillaceus* based on known cluster blast.

**Table 3:** Secondary metabolite cluster of *Streptomyces* sp. VITGV38 using antiSMASH 6.0.

S. No.	Region	Type	Most similar known cluster	Similarity (%)
1	Region 2.1	NRPS	Coelichelin	100
2	Region 2.2	RiPP-like	Informatipeptin	42
3	Region 2.3	NRPS	Coelibactin	100
4	Region 2.4	Terpene	Hopene	100
5	Region 2.5	Lanthipeptide class III	SapB	100
6	Region 3.1	NRPS, T1PKS	α-lipomycin	72
7	Region 4.1	NRPS-like, T1PKS, siderophore	α-lipomycin	31
8	Region 4.2	Terpene	Geosmin	100
9	Region 4.3	RiPP-like	—	
10	Region 4.4	T1PKS, NRPS-like, prodigiosin	Undecylprodigiosin	100
11	Region 5.1	Siderophore	Ficellomycin	3
12	Region 6.1	T2PK	Spore pigment	66
13	Region 6.2	Terpene	Abaflavenone	100
14	Region 6.3	RRE containing	Naphthomycin A	9
15	Region 9.1	Butyrolactone	Prejadomycin/Rabelomycin/	10
16	Region 10.1	T2PK	Prejadomycin/Rabelomycin/	27
17	Region 13.1	Terpene	—	
18	Region 14.1	RiPP-like	—	
19	Region 16.1	Phenazine	Iomofungin	34
20	Region 17.1	Siderophore	Desferrioxamin B/	83
21	Region 18.1	Melanin	Melanin	60
22	Region 24.1	Ectoine	Ectoine	100
23	Region 26.1	T3PKS	Herboxidiene	8
24	Region 26.2	T3PKS	Germicidin	10%
25	Region 26.3	Terpene	Carotenoid	45
26	Region 26.4	Indole	5-isoprenylindole-3-carboxylate β-D-glycosyl ester	23

NRPS, non-ribosomal peptide synthetase; T1PKS, type 1 polyketide synthases; T2PKS, type 2 polyketide synthases; T3PKS, type 3 polyketide synthases; RiPP, ribosomally synthesized and post-translationally modified peptides; RRE, RiPP recognition element.

The genetic similarity and organization of the biosynthetic gene clusters (BGCs) were analyzed using the “Minimum Information about a Biosynthetic Gene Cluster” (MIBiG) database. The BGC from *Streptomyces* sp. VITGV38 showed a similarity score of 0.30 with the isorenieratene—producing *Streptomyces argillaceus* (Figure 8C). A similarity score of 0.34 was observed with a β-carotene-producing uncultured bacterium, and a score of 0.32 was linked to a carotenoid BGC in *Streptomyces avermitilis*. These findings, along with other examples illustrated in the figure highlight the genetic relatedness of the carotenoid biosynthesis pathways across different *Streptomyces* species, suggesting a conserved evolutionary strategy for producing similar secondary metabolites across diverse bacterial taxa. The above BGC size is 24.01 kb. This is located at 676,310 to 700,326 nt. It contains up to eight related genes (Figure 8D), including both core biosynthetic, and regulatory genes which were predicted to be involved in isorenieratene biosynthesis. Based on the MIBiG analysis, the main genes in this cluster were 2Fe-2S ferredoxin, phytoene/squalene synthase, phytoene desaturase, geranylgeranyl pyrophosphate synthase, RNA polymerase Sigma-24 subunit, lycopene cyclase, methyltransferase, and isorenieratene synthase. Notably, the Pfam domain analysis revealed that more than 15 genes might contribute to isorenieratene biosynthesis (Figure 8E). A total of eight genes associated with the biosynthesis of carotenes are identified in this BGC and are listed in Table 4.

**Table 4:** Genes associated the biosynthesis of psi, psi-carotene (carotenes) are identified in this BGC.

Identifiers	Position	Product
crtaV	1–1,017 (−)	2Fe-2S ferredoxin
crtaB	1,014–2045 (−)	phytoene_/_squalene_synthase
crtaI	2042–3,586 (−)	phytoene desaturase
crtaE	3,583–4,974 (−)	geranylgeranyl pyrophosphate synthase
crtaQ	5,310–5,930 (+)	RNA_polymerase sigma-24_subunit
crtaY	6,172–7,362 (+)	lycopene_cyclase
crtaT	7,418–8,152 (+)	Methyltransferase
crtaU	8,149–9,777 (+)	isorenieratene synthase

### Carotenoid biosynthesis pathways

**Figure 9:**
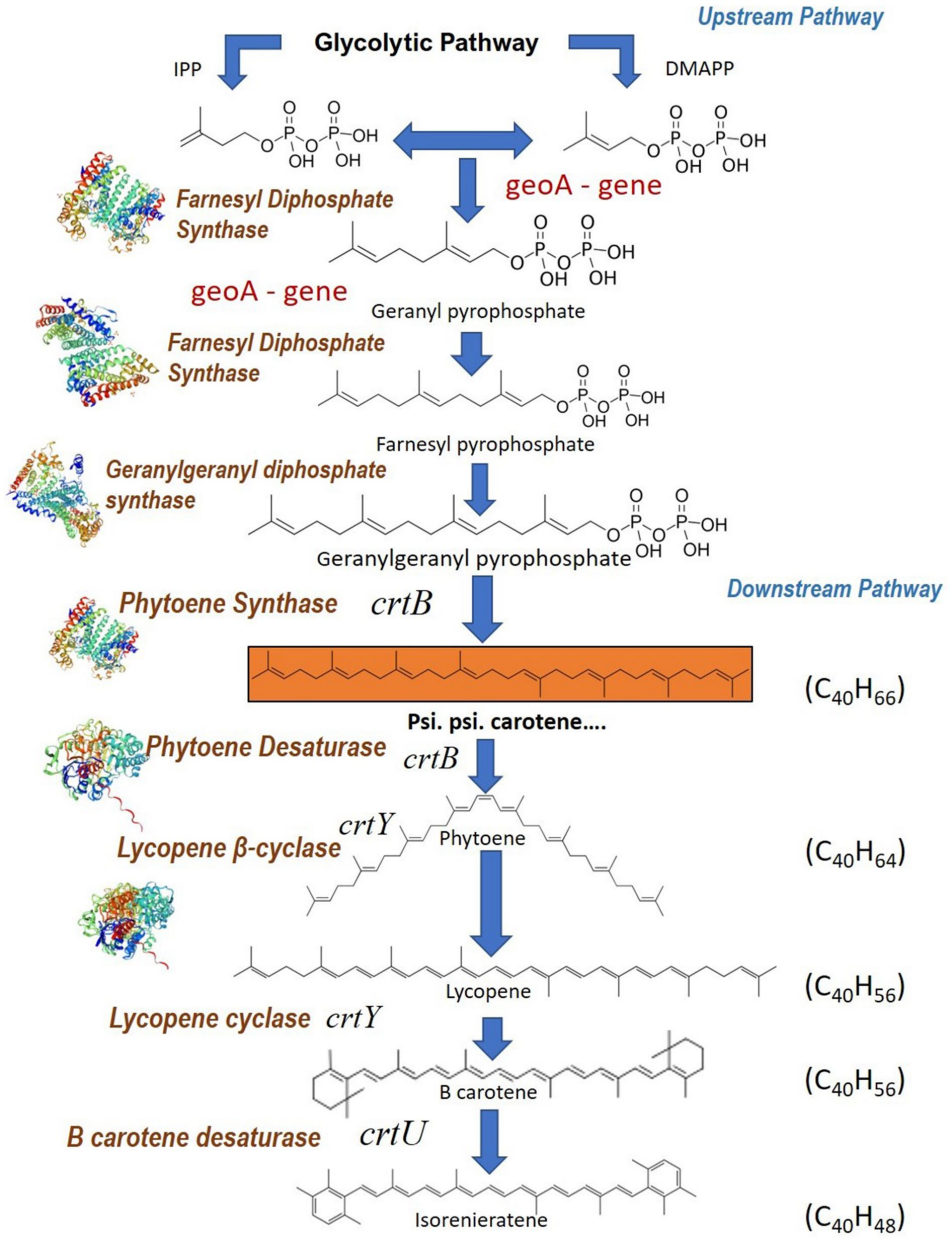
Psi, psi-carotene biosynthetic pathway of *Streptomyces* sp. VITGV38 and functions of the carotenoid biosynthesis genes associate with the compound. Inferred crt biosynthetic pathway leading to the formation of isoneriatene in *Streptomyces* sp. VITGV38. The enzyme encoded by each of the genes is also specified.

Carotenoid biosynthesis pathways in microorganisms comprise two distinct pathways viz. (i) upstream pathway and (ii) downstream pathway. The upstream pathway is directed at synthesizing the common precursors of carotene like isopentyl diphosphate (IPP) and dimethyl diphosphate (DMAPP) (Ma et al., 2016). The downstream pathway includes several enzymes that catalyze reactions that convert precursors to different kinds of carotenoids (Figure 9). The genes identified and analyzed in BGC in our analysis fall in the downstream pathway. The biosynthesis of carotenes starts with the conversion of DMAPP to geranyl diphosphate and subsequently into farnesyl diphosphate by the enzyme farnesyl diphosphate synthase. Farnesyl diphosphate is metabolically converted to geranylgeranyl diphosphate (GGPP) by the enzyme geranylgeranyl diphosphate synthase which is present in the gene clusters. Then the enzyme phytoene synthase catalyzes the conversion of GGPP to psi, psi-carotene.

## Discussion

The identification of *Streptomyces* sp. VITGV38 as an endophyte in tomato plants underscores the potential of endophytic actinomycetes as reservoirs of novel bioactive compounds. Previous studies by Veilumuthu and Christopher (2022a, 2022b, 2022c) documented the isolation of 240 actinomycete strains from tomato plants across various locations in Tamil Nadu, with each isolate designated under the VITGV series. Among these, VITGV38 was identified as a promising strain and subsequently genome-sequenced, with its sequence submitted to NCBI (Veilumuthu et al., 2023). These observations provide valuable insights into the ecological role of *Streptomyces* sp. VITGV38 as an endophyte, furthering our understanding of its potential contributions to plant health and microbial interactions. The distinct colony morphologies, mycelial formation, and spore structures suggest a metabolically versatile strain capable of producing secondary metabolites. Furthermore, preliminary antimicrobial screening of the crude extract reinforced the well-documented role of *Streptomyces* in natural product biosynthesis, emphasizing its potential biotechnological applications.

The *Streptomyces* sp. VITGV38 strain exhibited the formation of small clumps in liquid culture from the seventh day of incubation. By the 15th day at 30°C, in starch casein agar (SCA) for 7 days visible cell masses had developed within the flask. The strain demonstrated active production of secondary metabolites, as indicated by the presence of dark purple pigments in the culture medium. Morphological observations revealed an inward-growing primary mycelium, while secondary mycelium extended outward. Similar patterns of clump formation in *Streptomyces* species have been documented, including *Streptomyces olidensis*, as reported by Giudici et al. (2004). The observed morphological characteristics and pigment production suggest active biosynthesis, reinforcing the potential of *Streptomyces* sp. VITGV38 for secondary metabolite production.

GC-MS analysis of the ethyl acetate culture extract revealed 45 distinct peaks, indicative of a diverse metabolite profile. Among these, psi, psi-carotene was detected at a retention time of 25.094 min, constituting 1.26% of the total crude extract (Figure 4). These findings align with previous studies, such as the work of Al-Dhabi et al. (2020), who reported psi, psi-carotene at RT 19.9503 with an area percentage of 0.33% in *Streptomyces* sp. strain Al-Dhabi-97, isolated from marine environments in Saudi Arabia. Our results suggest that *Streptomyces* sp. VITGV38 demonstrates a higher yield of psi, psi-carotene compared to the previously reported marine strain, indicating its potential as a promising source for microbial carotenoid production. To evaluate the production efficiency of psi, psi-carotene in *Streptomyces* sp. VITGV38, we compared its yield with other reported microbial sources. The 1.26% yield observed in our study is notably higher than several previous reports, suggesting that endophytic *Streptomyces* strains may harbor enhanced metabolic pathways for carotenoid biosynthesis. Further optimization of fermentation conditions and genetic studies could enhance yield, making this strain a potential candidate for industrial applications.

This study presents the identification and characterization of *Streptomyces* sp. VITGV38 is an endophytic strain that produces the pigment psi, psi-carotene. Our findings have several implications and open new avenues for research in microbial genomics and natural product discovery. The endophytic nature of *Streptomyces* sp. VITGV38 aligns with previous research showing that endophytes play crucial roles in the physiology of host plants, offering benefits such as enhanced growth, defense mechanisms, and stress resistance. Our study extends this understanding by demonstrating that *Streptomyces* sp. VITGV38 not only supports plant health but also has significant biotechnological potential due to its pigment production. Through whole genome sequencing and bioinformatics analysis, we identified 26 BGCs within the 8.27 Mb genome of *Streptomyces* sp. VITGV38, including five terpene gene clusters. A similar result was reported in *Streptomyces* sp. VITGV100 which has 35 BGCs within the genome size is 8.08 Mb is reported by Veilumuthu and Christopher (2022c) The identification of psi, psi-carotene, confirmed by GCMS analysis and comparison with the NIST mass spectrophotometry database, represents a novel discovery for the *Streptomyces* genus. This is the first report of an endophytic *Streptomyces* species producing psi, psi-carotene, highlighting the metabolic diversity and unique biosynthetic capabilities of this strain. Interestingly, the biosynthesis of psi, psi-carotene in *Streptomyces* parallels similar reports of other carotenoids, such as psi, psi-carotene, produced by marine *Streptomyces* species. For instance, *Streptomyces* sp. strain Al-Dhabi-97, isolated from marine environments in Saudi Arabia, has been shown to produce carotenoids (Al-Dhabi et al., 2020).

The biosynthetic gene cluster (BGC) analysis of *Streptomyces* sp. VITGV38 revealed the presence of key genes involved in carotenoid biosynthesis, including phytoene synthase (crtB), phytoene desaturase (crtI), geranylgeranyl pyrophosphate synthase (crtE), lycopene cyclase (crtY), and isorenieratene synthase (crtU). These genes are fundamental in the conversion of precursor molecules into carotenoids, highlighting the strain’s potential for the biosynthesis of industrially relevant pigments. The genomic uniqueness of *Streptomyces* sp. VITGV38 is evident from its genetic divergence from other known carotenoid biosynthetic clusters. While it shares a 62% overall similarity with known pathways (Figure 8B), the presence of unique genetic elements suggests potential for carotenoid pigment production. To evaluate the uniqueness of this pathway, a comparative genomic analysis was performed using the Minimum Information about a Biosynthetic Gene Cluster (MIBiG) database, which revealed moderate similarity to previously reported bacterial carotenoid biosynthetic clusters (Figures 8C,D). The highest BGC similarity score (0.34) was observed with the β-carotene biosynthetic cluster from an uncultured bacterium in the environmental host of *Ralstonia metallidurans* (Craig et al., 2009), followed by isorenieratene biosynthesis in *Streptomyces argillaceus* (0.30) (Becerril et al., 2018) and carotenoid biosynthesis in *Streptomyces avermitilis* (0.32) (Omura et al., 2001). Despite these similarities, *Streptomyces* sp. VITGV38 harbors a distinct combination of genetic elements, particularly the crtU and crtT genes, which are associated with the isorenieratene biosynthesis pathway. The crtU gene plays a crucial role in catalyzing the conversion of lycopene into isorenieratene, a key carotenoid with unique structural modifications (Iftime et al., 2016). Furthermore, the crtT gene encodes a methyltransferase, potentially modifying the carotenoid backbone, which may influence pigment properties and stability (Lee et al., 2001). The genome sequencing and annotation of *Streptomyces* sp. VITGV38 provided an in-depth bioinformatic prediction of its secondary metabolite potential, which includes the complete set of genes for the biosynthesis of psi, psi carotene (lycopersene). The identification of key biosynthetic genes responsible for carotenoid synthesis not only establishes this strain as an untapped resource for natural pigment production but also lays the groundwork for future metabolic engineering efforts.

The genomic data revealed that the identified BGCs in *Streptomyces* sp. VITGV38 includes key enzymes involved in the carotenoid biosynthesis pathway, such as phytoene/squalene synthase, phytoene desaturase, and lycopene cyclase. The presence of these enzymes suggests a well-conserved biosynthetic pathway for carotenoids, which includes the conversion of common precursors like IPP and DMAPP to final carotenoid products. Two key biosynthetic routes exist for the production of IPP and DMAPP in nature: the mevalonate (MVA) pathway and the methylerythritol phosphate (MEP) pathway. The MVA pathway, initially studied by Block, Lynen, and Popjak, was originally thought to be the only source of isoprenoid precursors in animals and yeast, contributing to terpenoid synthesis, including carotenoids. However, it is now understood that both pathways play essential roles across different biological systems is studied by Moise et al. (2014) and Fimognari and Rodwell (1970).

This pathway has been well-documented in other microorganisms. In *Zymomonas mobilis*, *Methylobacterium fujisawaense*, *Escherichia coli*, and *Alicyclobacillus acidoterrestris*, the MEP pathway has been extensively studied by Schwender et al. (1996), demonstrating its role in isoprenoid precursor synthesis, which is crucial for carotenoid production. Similarly, in cyanobacteria such as *Synechocystis* sp. PCC 6803, carotenoid biosynthesis predominantly occurs via the MEP pathway, contributing to the production of pigments like β-carotene and zeaxanthin were reported by Schwender et al. (1996). The yeast *Saccharomyces cerevisiae* employs the MVA pathway for isoprenoid biosynthesis was reported by Shiba et al. (2007), which has been utilized in metabolic engineering to produce high levels of β-carotene. In contrast, *Bacillus subtilis* also utilizes the MEP pathway for carotenoid production as reported by Krubasik and Sandmann (2000), particularly under oxidative stress conditions, where carotenoids like C30 are produced to provide protective functions. Grob et al. (1961) reported the synthesis of psi, psi-carotene from [C_14_] geranylgeranyl pyrophosphate by a *Neurospora crassa* enzyme system. Phytoene is generally regarded as the first C_40_ carotenoid, although psi, psi-carotene (dihydro phytoene) has been proposed as a possible intermediate before phytoene (Arumugam et al., 2023). Further phytoene is converted to lycopene by the action of phytoene synthase resulting in four double bonds (Arrach et al., 2001; Martínez-Cámara et al., 2021). β-carotene is finally produced from lycopene by the action of lycopene cyclase (Schumann et al., 1996; Xin et al., 2021).

Psi, psi-carotene, a stable and safe triterpenoid, has been recognized for its diverse applications. The acquisition of pure psi, psi-carotene from natural sources holds significant importance for fundamental research endeavors. Hydroxy psi, psi-carotene shows antiproliferative activity against various tumor cells such as HeLa, PC3, HepG2, Hep3B, IHH (Hurtado-Díaz et al., 2019). Villasenor et al. (1993) isolated and investigated the antimutagenic activity of psi, psi-carotene from *Carmona retusa*. It shows 69% of activity against mutagenic activity. Similarly, the antiproliferative activity, anti-bacterial activity (Li et al., 2015; Pirmoradian and Hooshmand, 2019), pesticidal and insecticidal activity (Ebadollahi et al., 2015; Gupta, 2014), and cytotoxicity activity was evaluated. It shows very good pharmaceutical activity. In addition to its pharmacological properties, psi, psi-carotene also provides nutritional benefits. Tomatoes, a rich source of psi, psi-carotene, are known for their high content of vitamins, minerals, and other bioactive compounds that contribute to overall health and well-being. The biological importance of psi, psi-carotene lies in its multifaceted pharmacological activities, which make it a promising compound for various therapeutic and agricultural applications, as well as its potential contribution to human health and nutrition. The genetic insights gained from VITGV38 will facilitate synthetic biology approaches for improving carotenoid yield, optimizing biosynthetic pathways, and exploring its bioactive potential in pharmaceutical and industrial applications.

## Conclusion

The identification of *Streptomyces* sp. VITGV38 as an endophyte in tomato plants provides valuable insights into plant-microbe interactions. This strain was observed to produce pigmented compounds, including psi, psi-carotene, which constitutes a fraction of its crude extract. Genomic analysis revealed the presence of 26 distinct secondary metabolite gene clusters, including those associated with terpene biosynthesis and carotenoid production, particularly isorenieratene, a precursor in the psi, psi-carotene biosynthesis pathway. These findings suggest that *Streptomyces* sp. VITGV38 has the potential for microbial production of natural carotenoids, which can be widely utilized in biotechnology. Moreover, their industrial significance lies in their use as natural pigments, replacing synthetic colorants in food, cosmetics, and pharmaceuticals. While our findings indicate a promising biotechnological avenue, further studies on optimizing pigment production and evaluating its practical applications will be necessary to fully explore its industrial relevance. Notably, this is the first report of psi, psi-carotene biosynthesis from an endophytic *Streptomyces* species, expanding our understanding of microbial carotenoid production.

## Data Availability

The raw reads and assemblies for the isolate *Streptomyces* sp. VITGV38 is available at the NCBI—SRA portal under BioProject accession number PRJNA750621, BioSample accession number SAMN20475137, and SRA accession number SRS9634702.
