# Supplementary material for: Biosynthetic Pathway of psi, psi-Carotene from Streptomyces sp. VITGV38 (MCC 4869)
Source: Front Microbiol. 2025 Apr 1;16:1548894. doi: 10.3389/fmicb.2025.1548894 (PMC11998277; doi:10.3389/fmicb.2025.1548894)
Supplement: Supplementary file 1 [file Data_Sheet_1.docx]

Biosynthetic pathway of psi, psi-carotene from *Streptomyces* sp. VITGV38 (MCC 4869)

Veilumuthu Pattapulavar^1^, Sathiyabama Ramanujam^2^, Manoj Sekaran^3^, Rajasekaran Chandrasekaran^3^, Shweta Panchal^4^, and Godwin Christopher J^1^*

^1^Department of Biomedical Sciences, School of BioSciences and Technology, Vellore Institute of Technology, Vellore, Tamil Nadu 632014, India. 2

^2^Department of Science and Humanities, Karpagam Academy of Higher, Education, Coimbatore, Tamil Nadu, India- 641021.

^3^Department of Biotechnology, School of BioSciences and Technology, Vellore Institute of Technology, Vellore, Tamil Nadu 632014, India.

^4^Department of Integrative Biology, School of BioSciences and Technology, Vellore Institute of Technology, Vellore, Tamil Nadu 632 014, India.

**Correspondence:**Godwin Christopher J
godwinj@vit.ac.in

**Table** **S1** Compounds were detected from GCMS in the extract of Streptomyces sp. VITGV38

|  | **Chemical Compound** | **RT** | **Area %** | **Molecular formula** | **Molecular Weight** |
| --- | --- | --- | --- | --- | --- |
| 1 | Benzeneacetic acid | 11.655 | 1.49 | C_8_H_8_O_2_ | 136.15 |
| 2 | Indole | 12.821 | 0.65 | C_8_H_7_N | 117.15 |
| 3 | Benzoic acid, 2-amino- | 14.122 | 0.89 | C_7_H_7_NO_2_ | 137.14 |
| 4 | Acetamide, N-(2-phenylethyl)- | 15.866 | 1.26 | C_10_H_13_NO | 163.22 |
| 5 | Diethyl Phthalate | 16.286 | 0.60 | C_12_H_14_O_4_ | 222.24 |
| 6 | Tetradecanoic acid | 16.764 | 0.34 | C_14_H_28_O_2_ | 228.37 |
| 7 | 1,2-Benzenediol, 3,5-bis(1,1-dimethylethyl)- | 17.561 | 1.07 | C_14_H_22_O_2_ | 222.32 |
| 8 | 5-Octadecene, (E)- | 17.846 | 0.34 | C_18_H_36_ | 252.5 |
| 9 | 5-Methyl-2-phenylindolizine | 18.047 | 0.60 | C_15_H_13_N | 207.27 |
| 10 | n-Hexadecanoic acid | 18.853 | 4.92 | C_16_H_32_O_2_ | 256.42 |
| 11 | 3-Pyrrolidin-2-yl-propionic acid | 19.247 | 0.55 | C_7_H_13_NO_2_ | 143.18 |
| 12 | 7,9-Di-tert-butyl-1-oxaspiro (4,5) deca-6,9-diene-2,8-dione | 19.448 | 1.32 | C_17_H_24_O_3_ | 276.4 |
| 13 | Cyclo-(glycyl-l-leucyl) | 19.566 | 0.54 | C_8_H_14_N_2_O_2_ | 170.21 |
| 14 | 3,6-Diisopropylpiperazin-2,5-dione | 19.792 | 0.97 | C_10_H_18_N_2_O_2_ | 198.26 |
| 15 | Dibutyl phthalate | 19.977 | 4.82 | C_16_H_22_O_4_ | 278.34 |
| 16 | Tyramine, N-formyl- | 20.094 | 12.03 | C_9_H_11_NO_2_ | 165.19 |
| 17 | L-Proline, N-valeryl-, dodecyl ester | 20.346 | 0.65 | C_22_H_41_NO_3_ | 367.6 |
| 18 | 2,5-Piperazinedione, 3,6-bis(2-methylpropyl)- | 20.514 | 0.35 | C_12_H_22_N_2_O_2_ | 226.32 |
| 19 | N-Acetyltyramine | 20.681 | 1.67 | C_10_H_13_NO_2_ | 179.22 |
| 20 | 2,5-Cyclohexadien-1-one, 3,5-dihydroxy-4,4-dimethyl- | 20.883 | 1.54 | C_8_H_10_O_3_ | 154.16 |
| 21 | Pyrrolo[1,2-a] pyrazine-1,4-dione, hexahydro-3-(2-methylpropyl)- | 20.992 | 4.26 | C_11_H_18_N_2_O_2_ | 210.27 |
| 22 | Diethyldithiophosphinic acid | 21.168 | 1.33 | C_4_H_11_PS_2_ | 154.2 |
| 23 | l-Proline, N-allyloxycarbonyl-, propyl ester | 21.260 | 0.45 | C_12_H_19_NO_4_ | 241.28 |
| 24 | Octadecanamide | 21.722 | 0.48 | C_18_H_37_NO | 283.5 |
| 25 | Ethyl 5-chloro-2-nitrobenzoate | 22.133 | 1.29 | C_9_H_8_ClNO_4_ | 229.62 |
| 26 | p-Hydroxybiphenyl | 23.534 | 0.57 | C_12_H_10_O | 170.21 |

| 27 | o-Butyl O, O-diethyl phosphorothioate | 23.634 | 4.77 | C_8_H_19_O_3_PS | 226.28 |
| --- | --- | --- | --- | --- | --- |
| 28 | 3-Cyclohexylthiolane | 23.777 | 2.70 | C_10_H_18_S | 170.32 |
| 29 | Carbamic acid, methyl-, 3-methylphenyl ester | 24.171 | 1.80 | C_9_H_11_NO_2_ | 165.19 |
| 30 | 2,5-Piperazinedione, 3-benzyl-6-isopropyl- | 24.356 | 0.84 | C_14_H_18_N_2_O_2_ | 246.3 |
| 31 | Bicyclo[3.1.0]hex-3-en-2-one, 4-methyl-1-(1-methylethyl)- | 24.431 | 0.64 | C_10_H_14_O | 150.22 |
| 32 | Bis(2-ethylhexyl) phthalate | 24.582 | 1.79 | C_24_H_38_O_4_ | 390.6 |
| 33 | 1,1'-Biphenyl, 4-[(2-methyl-2-propenyl) oxy]- | 24.834 | 0.65 | C_16_H_16_O | 224.3 |
| 34 | Cyclo-(l-leucyl-l-phenylalanyl) | 24.960 | 0.39 | C_15_H_20_N_2_O_2_ | 260.329 |
| 35 | .psi.,.psi.-Carotene, 7,7',8,8',11,11',12,12',15,15'-decahydro- | 25.094 | 1.26 | C_40_H_66_ | 547 |
| 36 | Pyrrolo[1,2-a] pyrazine-1,4-dione, hexahydro-3-(phenylmethyl)- | 25.438 | 3.27 | C_14_H_16_N_2_O_2_ | 244.29 |
| 37 | 2-Amino-4-hydroxy-6-methylpyrimidine | 25.689 | 5.89 | C_5_H_7_N_3_O | 125.13 |
| 38 | 13-Docosenamide, (Z)- | 26.587 | 4.22 | C_22_H_43_NO | 337.6 |
| 39 | Acetamide, N-(4-methylphenyl)- | 27.518 | 1.46 | C_9_H_11_NO | 149.19 |
| 40 | 4-(p-Methoxyphenylazo)-m-phenylenediamine | 27.946 | 0.56 | C_13_H_14_N_4_O | 242.28 |
| 41 | Formamide, N-(2,4-diamino-1,6-dihydro-6-oxo-5-pyrimidinyl)- | 28.156 | 6.70 | C_5_H_7_N_5_O_2_ | 169.14 |
| 42 | Glycyl-L-tyrosine | 28.349 | 3.40 | C_11_H_14_N_2_O_4_ | 238.24 |
| 43 | Pyrimidine-2(1H)-thione, 4,4,6-trimethyl-1-(1-phenylethyl)- | 28.475 | 3.83 | C_15_H_20_N_2_S | 260.399 |
| 44 | l-Leucine, N-allyloxycarbonyl-, undec-10-enyl ester | 28.676 | 1.79 | C_21_H_37_NO_4_ | 367.5 |
| 45 | l-Proline, N-allyloxycarbonyl-, heptyl ester | 29.649 | 9.01 | C_16_H_27_NO_4_ | 297.39 |
